# Prognostic value of systemic inflammation response index in successfully recanalized acute large vessel occlusion stroke patients: a retrospective study

**DOI:** 10.3389/fneur.2026.1749452

**Published:** 2026-05-28

**Authors:** Wangyu Zhang, Congcong Zhou, Xuan Xu, Guomei Du, Shuqin Liang, Tong Xu, Han Zhao, Jiaou Chen

**Affiliations:** 1 Second Affiliated Hospital and Yuying Children’s Hospital of Wenzhou Medical University, Wenzhou, China; 2 Dongyang People’s Hospital, Dongyang, China; 3 Department of Cardiovascular and Thoracic Surgery, The Second Affiliated Hospital Zhejiang University School of Medicine, Hangzhou, China

**Keywords:** acute ischemic stroke, endovascular therapy, functional outcome, large vessel occlusion, systemic inflammation response index

## Abstract

**Background:**

Endovascular therapy (EVT), with or without intravenous thrombolysis, is the standard reperfusion treatment for acute large vessel occlusion stroke (ALVOS). However, nearly half of the patients still experience poor functional outcomes despite successful recanalization. This study aimed to investigate the relationship between baseline Systemic Inflammation Response Index (SIRI) and 90-day functional outcomes as well as hemorrhagic transformation (HT) in successfully recanalized ALVOS patients undergoing EVT from two stroke centers in Zhejiang, China.

**Methods:**

We conducted a dual-center, retrospective study enrolling 287 successfully recanalized ALVOS patients who underwent EVT. A good functional outcome was defined as a modified Rankin Scale (mRS) score of 0–2 at 90 days, while a poor outcome was defined as mRS 3–6. Univariate and multivariate logistic regression analyses were employed to explore the relationship between SIRI levels and 90-day neurological outcomes and HT. The predictive capability of SIRI for functional outcomes was assessed using receiver operating characteristic (ROC) curve analysis.

**Results:**

The mean age of the 287 included patients was 70.9 ± 13.0 years, with 168 (58.5%) being male. The mean SIRI level was 1.72 ± 1.92, which was significantly lower in the good outcome group (1.26 ± 1.10) than in the poor outcome group (2.22 ± 2.42, *p* < 0.001). The area under the ROC curve (AUC) for SIRI predicting a poor outcome was 0.63 (95% CI: 0.56–0.69, *P*<0.001). Incorporating SIRI into the age + NIHSS model significantly improved prognostic accuracy for 90 day outcomes (AUC increase from 0.76 to 0.79, ΔAUC = 0.035, *p* = 0.023). However, no significant association was found between SIRI levels and either hemorrhagic transformation (OR 1.08, 95% CI: 0.94–1.23, *p* = 0.29) or symptomatic intracranial hemorrhage (OR 1.12, 95% CI: 0.94–1.33, *p* = 0.22).

**Conclusion:**

A higher baseline SIRI level is associated with 90-day poor functional outcomes in successfully recanalized ALVOS patients after EVT, but not with hemorrhagic transformation. And adding SIRI to an age + NIHSS-based model significantly enhances prognostic discrimination. SIRI may serve as a readily available biomarker associated with early functional outcomes and could have potential value in prognostic assessment.

## Introduction

Stroke remains a leading cause of mortality and disability worldwide, with ischemic stroke accounting for approximately 80% of all cases (1–3). The cornerstone of acute ischemic stroke management is rapid revascularization to salvage the ischemic penumbra. Since the publication of five landmark randomized controlled trials (RCTs) in 2015 (4–8), with or without prior intravenous thrombolysis (IVT) (9), endovascular therapy (EVT), particularly mechanical thrombectomy (MT) (10), has become the standard of care for patients with ALVOS and is widely recommended in international guidelines.

Despite continuous innovations in EVT techniques and devices, leading to recanalization rates as high as 94% (11, 12), the clinical benefit has not been fully realized. Nearly half of the patients with successful recanalization (modified Thrombolysis in Cerebral Infarction (mTICI) grade 2b-3) do not achieve functional independence (mRS 0–2) at 90 days (13). This discrepancy between successful angiographic reperfusion and poor clinical outcome is termed “futile recanalization,” which occurs in 30–50% of cases (14). Therefore, identifying predictors of futile recanalization and poor functional outcomes has become a critical focus of stroke research.

Inflammation plays a pivotal role in the pathophysiology of cerebral ischemia and reperfusion injury. The Systemic Inflammation Response Index (SIRI), calculated as (neutrophil count × monocyte count)/lymphocyte count, is a novel integrative inflammatory biomarker first proposed by Qi et al. (15). It comprehensively reflects the balance between pro-inflammatory (neutrophils, monocytes) and immunomodulatory (lymphocytes) pathways. Due to its cost-effectiveness and routine availability from standard blood tests, SIRI has shown prognostic value in various inflammation-related diseases. Emerging evidence suggests a potential role for SIRI in stroke outcomes, yet its specific prognostic utility in ALVOS patients treated with EVT, particularly in Chinese populations, remains underexplored (15–17).

This study hypothesized that SIRI could serve as a reliable predictor for functional outcomes in successfully recanalized ALVOS patients. We aimed to investigate the association between baseline SIRI levels and 90-day functional outcomes, as well as hemorrhagic transformation, in a cohort of ALVOS patients from two stroke centers in Southern Zhejiang, China.

## Methods

### Patients

This was a dual-center, retrospective cohort study conducted at the Second Affiliated Hospital of Wenzhou Medical University and Taizhou First People’s Hospital. We screened all consecutive ALVOS patients who underwent EVT with successful recanalization between August 2020 and January 2024. The study was approved by the local Institutional Ethics Committees, which waived the requirement for written informed consent due to the retrospective nature of the analysis. Inclusion Criteria: (1) Age ≥ 18 years, (2) Acute ischemic stroke with large vessel occlusion confirmed by CTA, MRA, or DSA (involving the internal carotid artery, middle cerebral artery (M1 segment), anterior cerebral artery, vertebral-basilar artery, or posterior cerebral artery), (3) Pre-treatment National Institutes of Health Stroke Scale (NIHSS) score > 5, (4) Onset-to-puncture time (OTP) ≤ 24 h, (5) Undergone EVT according to the Chinese Guidelines for the Endovascular Treatment of Acute Ischemic Stroke (2023) with successful recanalization (mTICI 2b-3), (6) Availability of baseline complete blood count data before EVT. Exclusion Criteria: (1).

Intracranial hemorrhage on initial neuroimaging, (2) pre-stroke mRS > 2, indicating significant pre-existing disability, (3) concurrent active infection, inflammatory diseases, malignancy, or use of immunosuppressive medication, (4) Confirmed chronic vessel occlusion, (5) incomplete clinical or laboratory data.

### Data collection

Demographic data, past medical history (hypertension, atrial fibrillation, diabetes, stroke history, antiplatelet/anticoagulant use, smoking, alcohol consumption), baseline clinical characteristics (systolic and diastolic blood pressure, NIHSS score), and procedural details (IVT bridging therapy, OTP, onset-to-recanalization time (OTR), number of thrombectomy attempts, anesthesia type, occluded vessel) were collected from electronic medical records.

Laboratory parameters, including white blood cell count, neutrophil count, lymphocyte count, monocyte count, fibrinogen, creatinine, blood glucose, international normalized ratio (INR), and activated partial thromboplastin time (APTT), were measured from venous blood samples drawn at baseline before EVT. All hematological parameters were analyzed using an automated hematology analyzer (BC-7500).

### Outcome

Primary Outcome: The primary outcome was the 90-day functional status assessed via telephone interview using the mRS. A poor functional outcome was defined as mRS 3–6.

Secondary Outcome: The secondary outcome was the occurrence of hemorrhagic transformation (HT) on follow-up cranial CT or MRI within 36 h post-EVT. HT was defined as any hemorrhage within the ischemic territory not present on the initial scan. It was categorized according to the European Cooperative Acute Stroke Study II (ECASS II) criteria (18), into hemorrhagic infarction (HI-1, HI-2) or parenchymal hemorrhage (PH-1, PH-2). Symptomatic intracranial hemorrhage (sICH) was defined as any intracranial hemorrhage associated with a neurological deterioration of ≥4 points on the NIHSS from baseline, or leading to death.

All neuroimaging reviews and mRS assessments were performed by two experienced neurologists blinded to the patients’ clinical and laboratory data. Disagreements were resolved by consensus or adjudication by a third senior neurologist.

### Statistical analysis

Continuous variables with normal distribution were presented as mean ± standard deviation (SD) and compared using the Student’s *t*-test or one-way ANOVA. Non-normally distributed data were presented as median (interquartile range, IQR) and compared using the Mann–Whitney U test or Kruskal-Wallis test. Categorical variables were expressed as frequencies (%) and compared using the Chi-square test or Fisher’s exact test.

Multivariate logistic regression analyses were performed to evaluate the association between SIRI levels and poor functional outcomes. Results were reported as odds ratio (OR) with 95% confidence intervals (CI). Three progressively adjusted models were constructed: Model 1: Unadjusted. Model 2: Adjusted for covariates with age, NIHSS score at admission. Model 3: Adjusted for variables with *p* < 0.1 in univariate analysis (age, baseline NIHSS, baseline blood glucose, smoking history, hypertension, atrial fibrillation, bridging IVT, number of thrombectomy <4, OTP, OTR, anterior circulation occlusion, fibrinogen). Model 4: Adjusted for variables with *p* < 0.05 in univariate analysis (age, baseline NIHSS, baseline blood glucose, smoking history, hypertension, atrial fibrillation, bridging IVT, number of thrombectomy <4, OTP, OTR). SIRI was entered into the models both as a continuous variable (per 1-SD increase) and as a categorical variable (quartiles, with the first quartile as reference).

The discriminative ability of SIRI for predicting poor outcomes was assessed using ROC curve analysis, and the area under the curve (AUC) was calculated. Additional analyses compared the predictive performance of SIRI, NLR, and PLR using ROC curves and area under the curve (AUC) values. The incremental predictive ability of SIRI was assessed by comparing AUCs for the age + NIHSS model versus the age + NIHSS + SIRI model using the DeLong test. The optimal cut-off value was determined by the Youden’s index. Similar multivariate logistic regression models were used to analyze the association between SIRI and HT/sICH.

Multicollinearity among covariates (including age, NIHSS score at admission, SIRI, NLR, and PLR) was tested using variance inflation factor (VIF) and tolerance. A VIF < 5 and tolerance > 0.2 were considered acceptable indicators of the absence of multicollinearity.

All statistical analyses were performed using SPSS 26.0 (IBM Corp., Armonk, NY, United States). A two-sided *p*-value < 0.05 was considered statistically significant.

## Results

### Baseline characteristics

**Figure 1:**
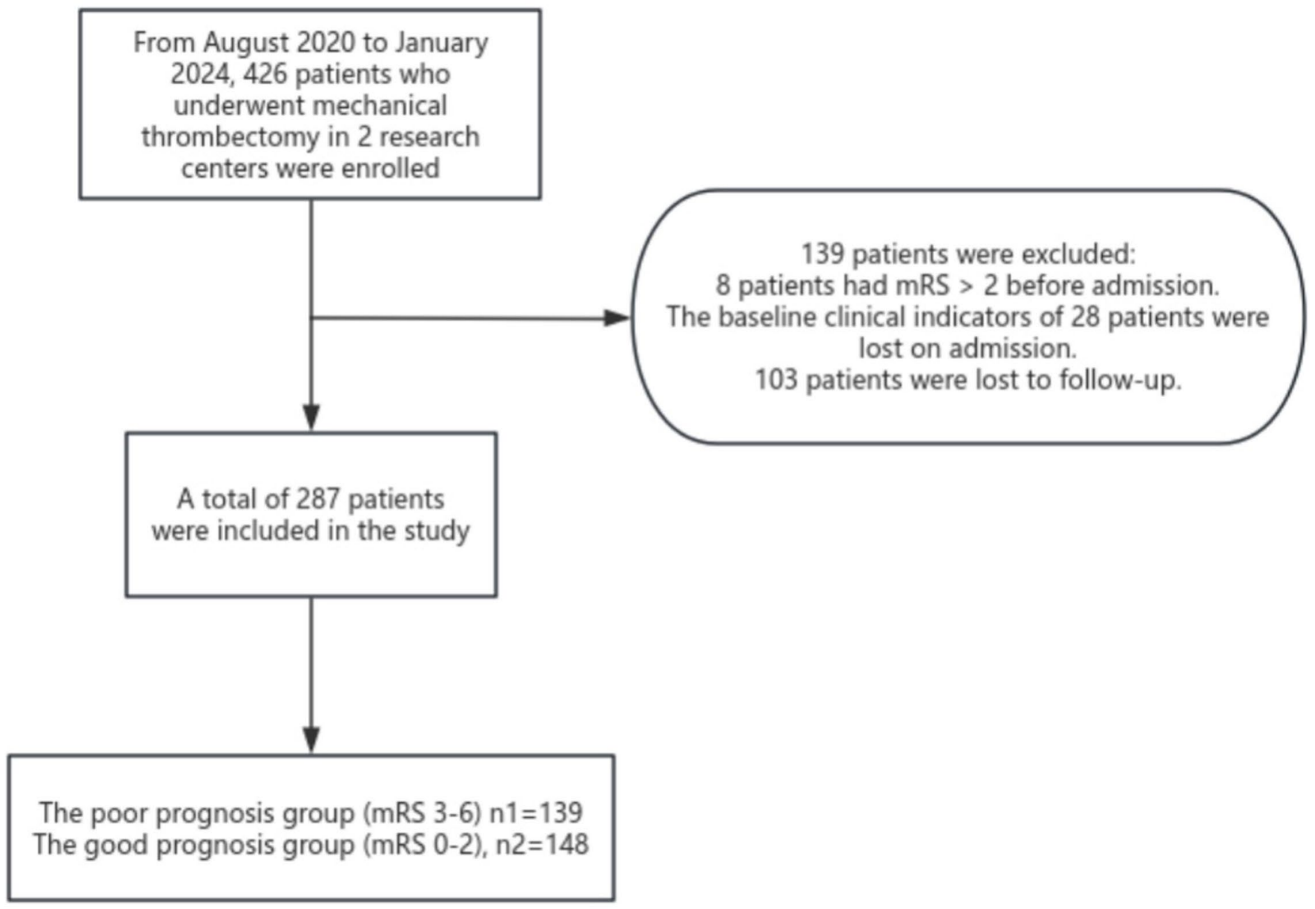
Flowchart of patient inclusion and analysis.

A total of 287 patients were included in the final analysis (Figure 1). The mean age was 70.9 ± 13.0 years, and 168 (58.5%) were male. Of these, 148 (51.6%) had a good outcome (mRS 0–2) and 139 (48.4%) had a poor outcome (mRS 3–6). The baseline characteristics of the patients stratified by functional outcome are summarized in Table 1. Patients in the poor outcome group were older, had higher baseline NIHSS scores, blood glucose levels, and a higher prevalence of atrial fibrillation and hypertension. They also had significantly higher SIRI levels (2.2 ± 2.4 vs. 1.3 ± 1.1, *p* < 0.001).

**Table 1:** Clinical characteristics of the 287 study subjects.

	Value
Demographics	
Male (%)	168(58.5%)
Age/years	70.9 ± 13.0
Past medical history	
Ischemic stroke (%)	62(21.6%)
Hemorrhagic stroke (%)	7(2.4%)
History of atrial fibrillation (%)	74(25.8%)
Atrial fibrillation after admission (%)	73(25.4%)
Hypertension (%)	180(62.7%)
Diabetes (%)	44(15.3%)
Cerebrovascular risk factors	
Previous smoking history (%)	55(19.2%)
Previous alcohol consumption (%)	32(11.1%)
Clinical characteristics on admission	
NIHSS score	14(10–18)
Systolic blood pressure (mmHg)	153.3 ± 23.1
Diastolic blood pressure (mmHg)	88.4 ± 14.8
White blood cell count (×10^9^/L)	7.7(6.3–9.6)
Neutrophil count ((×10^9^/L))	5.5(3.5–7.0)
Lymphocyte count (×10^9^/L)	1.8(1.7–2.5)
Monocyte count (×10^9^/L)	0.4(0.3–0.5)
SIRI	1.72 ± 1.92
INR	0.99(0.94–1.06)
APTT(sec)	32.9(30.4–35.6)
FIB(g/L)	3.1(2.7–3.6)
Blood glucose (mmol/L)	7.3(6.3–9.5)
Creatinine (mmol/L)	76(62–95)
OTP(min)	248(156–502)
OTR(min)	315(212–571)
Characteristics of reperfusion therapy	
Bridging therapy (%)	81(28.4%)
Local anesthesia (%)	192(66.9%)
Anterior circulation involvement (%)	252(87.8%)
< 4 thrombectomy (%)	248(86.4%)
Hemorrhagic transformation (%)	65(22.6%)
HI-1(%)	19(6.6%)
HI-2(%)	22(7.7%)
PH-1(%)	10(3.5%)
PH-2(%)	15(5.2%)

Values are n (%), median (IQR), or mean ± SD, unless otherwise noted. NIHSS, National Institutes of Health Stroke Scale; SIRI, Systemic Inflammation Response Index; INR, international normalized ratio; APTT, activated partial thromboplastin time; FIB, fibrinogen; OTP, onset-to-puncture time; OTR, onset-to-recanalization time; HI, hemorrhagic infarction; PH, parenchymal hemorrhage.

### SIRI and 90-day functional outcome

In univariate analysis (Table 2), SIRI was significantly associated with a poor outcome. This association remained significant after multivariate adjustment (Table 3). In the fully adjusted model (Model 4), for every 1-SD increase in SIRI, the odds of a good outcome decreased by 56% (OR 0.44, 95% CI 0.27–0.70, *p* = 0.001).

**Table 2:** Comparison of baseline indicators between the good and poor prognosis groups.

	Good outcome group mRS 0–2 (*n* = 148)	Poor outcome group mRS 3–6 (*n* = 139)	*p*-value
Demographics			
Male (%)	91(61.5%)	77(55.4%)	0.30
Age/years	66.72 ± 13.17	75.32 ± 11.22	<0.001^*^
Past medical history			
Ischemic stroke (%)	33(22.3%)	29(20.9%)	0.77
Hemorrhagic stroke (%)	4(2.7%)	3(2.2%)	0.77
History of atrial fibrillation (%)	35(23.6%)	39(28.1%)	0.39
Atrial fibrillation after admission (%)	28(18.9%)	45(32.4%)	0.009^*^
Hypertension (%)	83(56.1%)	97(69.8%)	0.016^*^
Diabetes (%)	21(14.2%)	23(16.5%)	0.58
Cerebrovascular risk factors			
Previous smoking history (%)	35(23.6%)	20(14.4%)	0.047^*^
Previous alcohol consumption (%)	17(11.5%)	15(10.8%)	0.85
Clinical characteristics on admission			
NIHSS score	11(8–16)	16(12–20)	<0.001^*^
Systolic blood pressure (mmHg)	152.08 ± 1.55	154.64 ± 24.77	0.35
Diastolic blood pressure (mmHg)	88.11 ± 13.80	88.65 ± 15.78	0.76
White blood cell count (×10^9^/L)	7.35(5.80–9.10)	8.00(6.60–10.10)	0.016^*^
Neutrophil count (×10^9^/L)	4.40(3.43–6.20)	5.50(3.7–7.7)	0.001^*^
Lymphocyte count (×10^9^/L)	1.90(1.33–2.70)	1.50(1.1–2.2)	0.003^*^
Monocyte count (×10^9^/L)	0.40(0.30–0.50)	0.40(0.30–0.50)	0.50
SIRI	1.26 ± 1.10	2.22 ± 2.42	<0.001^*^
INR	0.98(0.93–1.05)	1.01(0.95–1.09)	0.018^*^
APTT(sec)	32.5(30.2–35.5)	33.0(30.5–35.8)	0.30
FIB(g/L)	3.05(2.68–3.60)	3.20(2.82–3.71)	0.089
Blood glucose (mmol/L)	7.06(6.08–8.80)	7.60(6.65–10.15)	0.01^*^
Creatinine (mmol/L)	74(63–89)	78(59–102)	0.40
OTP(min)	228(153–427)	273(165–680)	0.038^*^
OTR(min)	276(201–470)	332(228–765)	0.033^*^
Characteristics of reperfusion therapy			
Bridging therapy (%)	52(35.1%)	29(20.9%)	0.007^*^
Local anesthesia (%)	104(70.3%)	88(63.3%)	0.21
Anterior circulation involvement (%)	125(84.5%)	127(91.4%)	0.074
< 4 thrombectomy (%)	126(91.1%)	112(80.6%)	0.005^*^

Values are n (%), median (IQR), or mean ± SD, unless otherwise noted. NIHSS, National Institutes of Health Stroke Scale; SIRI, Systemic Inflammation Response Index; INR, international normalized ratio; APTT, activated partial thromboplastin time; FIB, fibrinogen; OTP, onset-to-puncture time; OTR, onset-to-recanalization time; HI, hemorrhagic infarction; PH, parenchymal hemorrhage. *Marked represents *P* < 0.05, indicating statistical significance.

**Table 3:** Multivariate logistic regression analysis of SIRI levels and poor functional outcome at 90 days.

	Model 1	Model 3	Model 4
	OR(95%CI) *P*-value	OR(95%CI) *P*-value	OR(95%CI) *P*-value
SIRI	0.68(0.57–0.83) <0.001^*^	0.62(0.47–0.80) <0.001^*^	0.63(0.49–0.81) <0.001^*^
Quartile of SIRI			
Q1( < 0.6)	Reference	Reference	Reference
Q2(0.6–1.1)	0.86(0.44–1.68) 0.66	0.72(0.31–1.68) 0.45	0.76(0.34–1.71) 0.51
Q3(1.1–2.1)	0.60(0.31–1.17) 0.13	0.50(0.22–1.13) 0.094	0.53(0.23–1.18) 0.12
Q4( > 2.1)	0.32(0.16–0.63) 0.001^*^	0.22(0.09–0.54) <0.001^*^	0.25(0.11–0.58) 0.001^*^

Model 1, with no covariate adjustment. Model 3, adjusted for covariates with *P* < 0.1 (age, NIHSS score at admission, baseline blood glucose, smoking history, history of hypertension, diagnosis of atrial fibrillation after admission, receipt of bridging therapy, number of thrombectomy less than 4, OTP, OTR, anterior circulation, FIB). Model 4, adjusted for covariates with *P* < 0.05 (age, NIHSS score at admission, baseline blood glucose, history of smoking, history of hypertension, diagnosis of atrial fibrillation after admission, receipt of bridging therapy, number of thrombectomy less than 4, OTP, OTR). Multivariate Logistic regression analysis was used to calculate beta values and 95% confidence intervals. 95%CI, 95% confidence interval; OR, odds ratio; SIRI, Systemic inflammatory response index. *Marked represents *p* < 0.05, indicating statistical significance.

**Figure 2:**
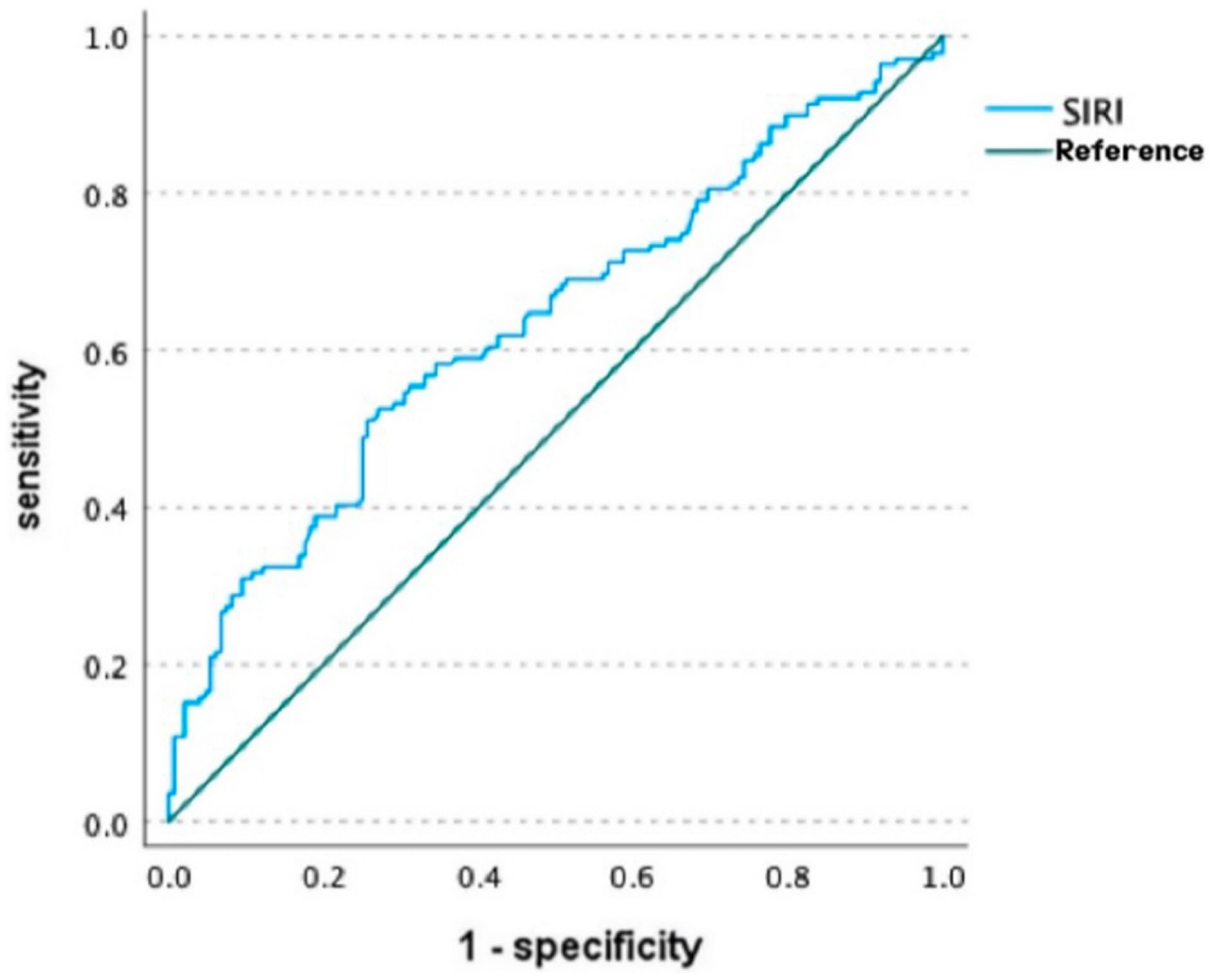
Association of SIRI levels with poor functional outcome at 90 days. SIRI, Systemic Inflammation Response Index; AUC = 0.63 (95% CI: 0.56–0.69, *p* < 0.001).

The ROC curve analysis (Figure 2) demonstrated that SIRI had a modest but significant predictive ability for poor outcomes, with an AUC of 0.63 (95% CI: 0.56–0.69, *P*<0.001). The optimal SIRI cut-off value was 1.47, yielding a sensitivity of 52.5% and a specificity of 73.0%.

### Comparative predictive performance of SIRI, NLR, and PLR

In ROC analysis, the base model with age + NIHSS yielded an AUC of 0.76 (95% CI: 0.70–0.82, *p* < 0.001). Adding SIRI to the model increased the AUC to 0.79 (95% CI: 0.74–0.85, *p* < 0.001), with ΔAUC = 0.035 (*p* = 0.023), indicating a statistically significant improvement but modest improvement in predictive ability (Supplementary Figure S1).

**Figure 3:**
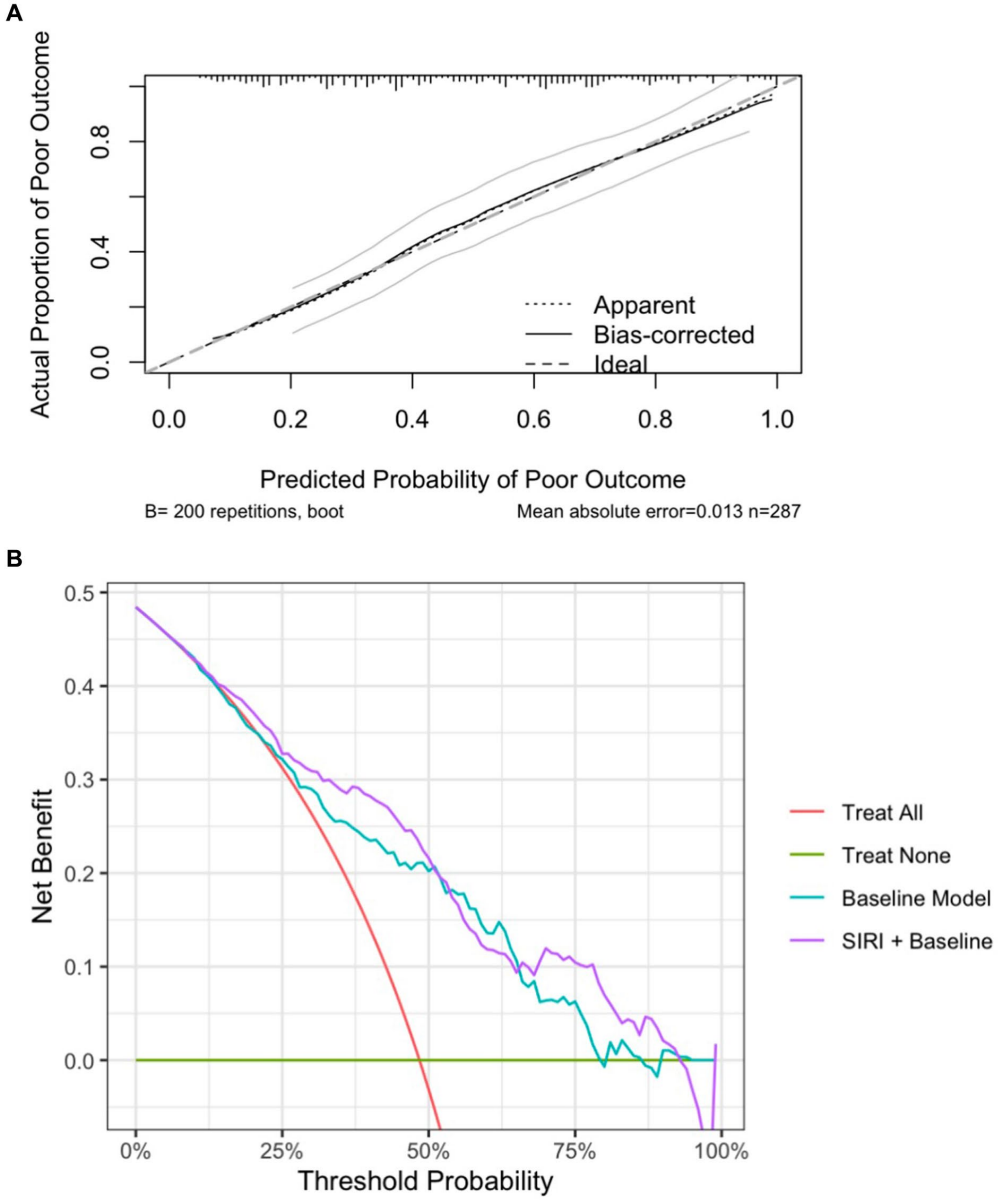
**(A)** Calibration curve for SIRI-added model. **(B)** Decision curve analysis (DCA).

Furthermore, the calibration of the SIRI-added model was assessed using 200 bootstrap resamples. The calibration curve (Figure 3) showed excellent agreement between the predicted and observed probabilities of poor outcome, with a mean absolute error (MAE) of 0.013. To evaluate clinical utility, Decision Curve Analysis (DCA) was performed. The results indicated that the inclusion of SIRI provided a higher net benefit compared with the baseline model (Age + NIHSS) and the two default strategies (treat-all and treat-none) across a wide range of threshold probabilities (approximately 15–90%), demonstrating the clinical value of SIRI in risk stratification (Figure 3B).

In univariate and multivariable logistic regression analyses (Supplementary Table S3), SIRI, NLR, and PLR were all significantly associated with poor 90 day functional outcome. In direct ROC comparisons (Supplementary Figure S2), the AUCs of SIRI and NLR did not differ significantly (ΔAUC = 0.014, *p* = 0.41), whereas both were superior to PLR (*p* < 0.05). When all three indices were included in Model 4 (Supplementary Figure S3), SIRI yielded the highest AUC (0.83), exceeding NLR (0.82) and PLR (0.82).

### SIRI and hemorrhagic transformation

Sixty-five patients (22.6%) developed HT. The distribution was: HI1 in 19 (6.6%), HI2 in 22 (7.7%), PH1 in 10 (3.5%), and PH2 in 15 (5.2%) patients. Univariate analysis showed no significant difference in SIRI levels between the HT and non-HT groups (*p* = 0.056) (Table 4). In multivariate logistic regression analysis (Table 5), SIRI was not independently associated with HT (adjusted OR per SD increase: 1.08, 95% CI: 0.94–1.23, *p* = 0.29) or sICH (adjusted OR per SD increase: 1.12, 95% CI: 0.94–1.33, *p* = 0.22) (Tables 6, 7).

**Table 4:** Univariate comparison between the hemorrhagic transformation group and the non-hemorrhagic transformation group.

	HT group (*n* = 65)	Non-HT group (*n* = 222)	*P*-value
Demographics			
Male (%)	33 (50.8%)	135 (60.8%)	0.15
Age/years	73.48 ± 12.8	70.13 ± 13.0	0.067
Past medical history			
Ischemic stroke (%)	17 (26.2%)	45 (20.3%)	0.31
Hemorrhagic stroke (%)	1 (1.5%)	6 (2.7%)	0.59
History of atrial fibrillation (%)	22 (33.8%)	52 (23.4%)	0.091
Atrial fibrillation after admission (%)	16 (24.6%)	57 (25.7%)	0.86
Hypertension (%)	39 (60.0%)	141 (63.5%)	0.61
Diabetes (%)	10 (15.4%)	34 (15.3%)	0.99
Previous medication history			
Antiplatelet agents (%)	4 (6.2%)	20 (9.0%)	0.47
Anticoagulants (%)	14 (21.5%)	31 (14.0%)	0.14
Cerebrovascular risk factors			
Previous smoking history (%)	11 (16.9%)	44 (19.8%)	0.60
Previous alcohol consumption (%)	10(15.4%)	22 (9.9%)	0.22
Clinical characteristics on admission			
NIHSS score	15 (12–19)	13 (9–18)	0.025^*^
Systolic blood pressure (mmHg)	151 ± 24	154 ± 23	0.39
Diastolic blood pressure (mmHg)	87 ± 14	89 ± 15	0.41
White blood cell count (×10^9^/L)	7.8 (6.5–10.5)	7.7 (6.3–9.4)	0.44
Neutrophil count (×10^9^/L)	5.2 (3.9–7.7)	4.9 (3.5–6.9)	0.21
Lymphocyte count (×10^9^/L)	1.5 (1.1–2.2)	1.8 (1.2–2.5)	0.051
Monocyte count (×10^9^/L)	0.4 (0.3–0.5)	0.4 (0.3–0.5)	0.42
SIRI	2.13 ± 2.52	1.60 ± 1.69	0.056
INR	1.0 (0.9–1.1)	1.0 (0.9–1.1)	0.39
APTT(sec)	33.0 (30.3–36.2)	32.8 (30.4–35.5)	0.94
FIB(g/L)	3.1 (2.7–3.5)	3.2 (2.7–3.7)	0.43
Blood glucose (mmol/L)	7.9 (6.6–10.5)	7.2 (6.3–9.2)	0.069
Creatinine (mmol/L)	84.3 (63.0–99.5)	74.0 (61.0–93.3)	0.19
OTP(min)	345 (204–726)	236 (153–430)	0.005^*^
OTR(min)	430 (252–790)	285 (200–490)	0.004^*^
Characteristics of reperfusion therapy			
Bridging therapy (%)	15 (23.1%)	66 (29.7%)	0.30
Local anesthesia (%)	46 (70.8%)	146 (65.8%)	0.45
Anterior circulation involvement (%)	62 (95.4%)	190 (85.6%)	0.034^*^
< 4 thrombectomy (%)	49 (75.4%)	119 (89.6%)	0.003^*^
Pneumonia during hospitalization	36 (55.4%)	84 (37.8%)	0.012^*^

Values are n (%), median (IQR), or mean ± SD, unless otherwise noted. NIHSS, National Institutes of Health Stroke Scale; SIRI, Systemic Inflammation Response Index; INR, international normalized ratio; APTT, activated partial thromboplastin time; FIB, fibrinogen; OTP, onset-to-puncture time; OTR, onset-to-recanalization time; HI, hemorrhagic infarction; PH, parenchymal hemorrhage. *Marked represents *P* < 0.05, indicating statistical significance.

**Table 5:** Multivariate logistic regression analysis of SIRI levels and hemorrhagic transformation.

	Model 1	Model 3	Model 4
	OR(95%CI) *P*-value	OR(95%CI) *P*-value	OR(95%CI) *P*-value
SIRI	1.13 (0.99–1.29) 0.66	1.08 (0.94–1.23) 0.29	1.07 (0.93–1.23) 0.36
Quartile of SIRI			
Q1(<0.6)	Reference	Reference	Reference
Q2(0.6–1.1)	1.56 (0.67–3.63) 0.31	1.80 (0.74–4.37) 0.19	1.84 (0.75–4.48) 0.18
Q3(1.1–2.1)	1.92 (0.83–4.43) 0.13	2.05 (0.85–4.93) 0.11	2.09 (0.87–5.04) 0.10
Q4(>2.1)	2.13 (0.94–4.86) 0.07	2.06 (0.85–5.00) 0.11	2.08 (0.85–5.07) 0.11

Model 1, with no covariate adjustment. Model 3, adjusted for covariates with *P* < 0.1 (age, NIHSS score at admission, baseline blood glucose, smoking history, history of hypertension, diagnosis of atrial fibrillation after admission, receipt of bridging therapy, number of thrombectomy less than 4, OTP, OTR, anterior circulation, FIB). Model 4, adjusted for covariates with *P* < 0.05 (age, NIHSS score at admission, baseline blood glucose, history of smoking, history of hypertension, diagnosis of atrial fibrillation after admission, receipt of bridging therapy, number of thrombectomy less than 4, OTP, OTR). Multivariate Logistic regression analysis was used to calculate beta values and 95% confidence intervals. 95%CI, 95% confidence interval; OR, odds ratio; SIRI, Systemic inflammatory response index.

**Table 6:** Univariate analysis of clinical data between patients in the symptomatic intracranial hemorrhage group and the asymptomatic intracranial hemorrhage group.

	sICH group n5 = 25	non-sICH group n6 = 262	*P*-value
Characteristics of the population			
Male (%)	14 (56.0%)	154 (58.8%)	0.79
Age/years	75.3 ± 13.1	70.5 ± 12.9	0.76
Past medical history			
Ischemic stroke (%)	6 (24.0%)	56 (21.4%)	0.76
Hemorrhagic stroke (%)	1 (4.0%)	6(2.3%)	0.62
History of atrial fibrillation (%)	11 (44.0%)	63 (24.0%)	
Atrial fibrillation after admission (%)	6 (24.0%)	67 (25.6%)	0.86
Hypertension (%)	12 (48.0%)	168 (64.1%)	0.11
Diabetes (%)	2 (8.0%)	42(16.0%)	0.44
Previous medication history			
Antiplatelet agents (%)	3(12.0%)	21(8.0%)	0.75
Anticoagulants (%)	7(28.0%)	38(14.5)	0.14
Cerebrovascular risk factors			
Previous smoking history (%)	5(20%)	50(19.1%)	1.0
Previous alcohol consumption (%)	6 (24.0%)	26 (9.9%)	0.71
Clinical characteristics on admission			
NIHSS score	16 (12–19)	14 (9–18)	0.064
Systolic blood pressure (mmHg)	148.0 ± 26.0	153.8 ± 22.9	0.23
Diastolic blood pressure (mmHg)	87.6 ± 15.8	88.5 ± 15.7	0.70
White blood cell count (×10^9^/L)	8.5 (7.1–11.1)	7.6(6.2–9.4)	0.071
Neutrophil count ((×10^9^/L))	6.2(3.8–7.7)	4.9(3.5–6.9)	0.26
Lymphocyte count (×10^9^/L)	1.8(1.2–2.6)	1.8(1.2–2.5)	0.87
Monocyte count (×10^9^/L)	0.5(0.4–0.7)	0.4(0.3–0.5)	0.011^*^
SIRI	2.36 ± 2.58	1.66 ± 1.84	0.088
INR	1.01 (0.97–1.13)	0.99 (0.94–1.05)	0.074
APTT(sec)	35.2 (30.5–38.8)	32.8 (30.3–35.5)	0.12
FIB(g/L)	3.0 (2.3–3.2)	3.2 (2.7–3.7)	0.023^*^
Blood glucose (mmol/L)	7.9 (6.1–10.1)	7.3 (6.3–9.4)	0.73
Creatinine (mmol/L)	83.0 (61.7–103.5)	75.0 (61.8–94.3)	0.47
OTP(min)	333 (221–749)	241 (155–455)	0.046^*^
OTR(min)	460 (265–825)	310 (206–541)	0.041^*^
Characteristics of reperfusion therapy			
Bridging Therapy (%)	8 (32.0%)	73 (27.9%)	0.67
Local anesthesia (%)	17 (68.0%)	175 (66.8%)	0.90
Anterior circulation involvement (%)	24 (96.0%)	228 (87.0%)	0.32
< 4 thrombectomy (%)	17 (68.0%)	231 (88.2%)	0.012^*^
Pneumonia during hospitalization (%)	16 (64%)	104 (39.7%)	0.019^*^

Values are n (%), median (IQR), or mean ± SD, unless otherwise noted. NIHSS, National Institutes of Health Stroke Scale; SIRI, Systemic Inflammation Response Index; INR, international normalized ratio; APTT, activated partial thromboplastin time; FIB, fibrinogen; OTP, onset-to-puncture time; OTR, onset-to-recanalization time; HI, hemorrhagic infarction; PH, parenchymal hemorrhage. *Marked represents *P* < 0.05, indicating statistical significance.

**Table 7:** Multivariate logistic regression analysis of SIRI levels and symptomatic intracranial hemorrhage.

	Model 1	Model 3	Model 4
	OR(95%CI) *P*-value	OR(95%CI) *P*-value	OR(95%CI) *P-*value
SIRI	1.14 (0.97–1.34) 0.10	1.12 (0.94–1.33) 0.22	1.12 (0.94–1.33) 0.21
Quartile of SIRI			
Q1(<0.6)	Reference	Reference	Reference
Q2(0.6–1.1)	1.52 (0.41–5.63) 0.53	1.95 (0.48–7.90) 0.35	1.73 (0.44–6.79) 0.43
Q3(1.1–2.1)	1.59 (0.43–5.91) 0.49	1.96(0.49–7.85) 0.34	1.71 (0.44–6.69) 0.44
Q4(>2.1)	2.43 (0.71–8.28) 0.16	3.08 (0.79–12.09) 0.11	2.75 (0.72–10.49) 0.14

Model 1, with no covariate adjustment. Model 3, adjusted for covariates with *P* < 0.1 (age, NIHSS score at admission, baseline blood glucose, smoking history, history of hypertension, diagnosis of atrial fibrillation after admission, receipt of bridging therapy, number of thrombectomy less than 4, OTP, OTR, anterior circulation, FIB). Model 4, adjusted for covariates with *P* < 0.05 (age, NIHSS score at admission, baseline blood glucose, history of smoking, history of hypertension, diagnosis of atrial fibrillation after admission, receipt of bridging therapy, number of thrombectomy less than 4, OTP, OTR). Multivariate Logistic regression analysis was used to calculate beta values and 95% confidence intervals. 95%CI, 95% confidence interval; OR, odds ratio; SIRI, Systemic inflammatory response index.

### Subgroup analysis

A subgroup logistic regression analysis was conducted according to baseline NIHSS scores, dividing patients into mild to moderate stroke (NIHSS ≤ 15) and severe stroke (NIHSS > 15) groups (Supplementary Table S1). In the mild to moderate group, baseline SIRI was significantly associated with poor 90 day functional outcome across all models: Model 1 (OR = 0.68, 95% CI 0.57–0.83, *p* < 0.001), Model 2 (OR = 0.65, 95% CI 0.50–0.85, *p* = 0.001), Model 3 (OR = 0.63, 95% CI 0.48–0.83, *p* = 0.001), and Model 4 (OR = 0.63, 95% CI 0.47–0.85, *p* = 0.002). In contrast, among patients with severe stroke (NIHSS > 15), SIRI was significantly associated with outcome in Model 1 (OR = 0.56, 95% CI 0.35–0.90, *p* = 0.015) and Model 2 (OR = 0.52, 95% CI 0.30–0.90, *p* = 0.019), but the association was no longer statistically significant in Model 3 (OR = 0.66, 95% CI 0.17–1.16, *p* = 0.15) and Model 4 (OR = 0.60, 95% CI 0.34–1.06, *p* = 0.08). Outcome variable coded as 1 = good, 0 = poor, thus OR < 1 indicates higher risk.

### Multicollinearity analysis

The multicollinearity diagnostic results are shown in Supplementary Table S2. For age, NIHSS score, and SIRI, tolerance values were all > 0.9 and VIF values < 1.1, indicating no significant multicollinearity among these variables.

## Discussion

This dual-center retrospective study demonstrates that a higher baseline SIRI level is strongly associated with an increased risk of 90-day poor functional outcomes in successfully recanalized ALVOS patients treated with EVT. SIRI demonstrated a comparable predictive performance to NLR but significantly outperformed PLR in discriminating poor functional outcomes at 90 days. Moreover, the addition of SIRI to a base prediction model containing age and NIHSS showed a statistically significant but moderate increase in the AUC by 0.035 (*p* = 0.023), highlighting its incremental prognostic value. When all three markers were included in the fully adjusted model, SIRI yielded the highest AUC, suggesting that SIRI may integrate prognostic information more effectively than NLR or PLR in the post-recanalization setting. However, we found no significant association between SIRI and the risk of hemorrhagic transformation or symptomatic intracranial hemorrhage.

Our findings align with the growing body of evidence highlighting the critical role of systemic inflammation in stroke pathophysiology and recovery. Neutrophils are among the first responders to cerebral ischemia (19), and pre-procedural neutrophilia has been identified as an independent predictor of poor outcomes after EVT in anterior circulation (20) and basilar artery occlusion (21). Lymphocytes, crucial for inflammation resolution and repair (22), are often depleted in acute stroke due to physiological stress, and lymphopenia is associated with larger infarct volumes and early neurological deterioration (23). Monocytes contribute to tissue damage through the release of proteases like matrix metalloproteinase-9 (24). In the initial phase of the inflammatory response, peripheral monocytes and endothelial cells release tissue factor (25, 26), and upregulate the expression of plasminogen activator inhibitor-1 (PAI-1) in endothelial cells (27). This cascade of events may ultimately contribute to further injury within the ischemic brain tissue (28). While traditional ratios like NLR and PLR have been widely studied (29–33). SIRI integrates these three distinct immune pathways (34, 35), offering a more comprehensive reflection of the net inflammatory state (36). Our results confirm and extend previous work by Huang (37), suggesting that SIRI is a robust biomarker in the stroke context (38).

It is well-established that hemorrhagic transformation is an independent risk factor for both neurological deterioration and poor long-term prognosis after reperfusion therapy in patients with acute ischemic stroke (9, 39, 40). The lack of association between SIRI and HT/sICH is noteworthy in our study. While inflammation is known to disrupt the blood–brain barrier and contribute to HT (41, 42), our negative result suggests that the baseline systemic inflammatory status, as captured by a single SIRI measurement at baseline, may not be the primary driver of hemorrhagic complications post-EVT. This could be because HT is more strongly influenced by other factors, such as the extent of ischemic damage, reperfusion intensity, and procedural techniques. Alternatively, the dynamic changes in inflammation after reperfusion, which are not captured by a baseline value, might be more relevant to HT pathogenesis. Future studies with serial SIRI measurements are warranted to explore this hypothesis.

Subgroup analysis indicates that the prognostic association between baseline SIRI and 90 day outcomes was robust in patients with mild to moderate stroke across all adjustment levels, but was not sustained in severe stroke patients after full adjustment. In severe cases, the loss of statistical significance in Models 3 and 4 suggests that other strong prognostic determinants such as baseline neurological injury severity, major comorbidities, and procedural variables may dominate outcome prediction, thereby attenuating the measurable effect of SIRI. Conversely, in mild to moderate patients, systemic inflammatory response as captured by SIRI may play a proportionally larger role in influencing functional recovery, underscoring its potential clinical utility for prognostic stratification in this subgroup.

### Limitations

Our study has several limitations. First, its retrospective and dual-center design may introduce selection bias. Although we followed a standardized protocol for data collection, some patients were excluded due to missing laboratory data or loss to follow-up, which might affect the generalizability of our results. Second, we used a single baseline SIRI measurement, which does not account for dynamic inflammatory changes during and after EVT. Third, the sample size, particularly for the sICH subgroup analysis, was relatively small, potentially limiting the statistical power to detect a weaker association. Fourth, although we adjusted for numerous confounders, residual confounding from unmeasured factors (e.g., detailed infarct core volume) cannot be excluded. Fifth, This study included patients who underwent endovascular therapy within 24 h after stroke onset, which may have introduced clinical and biochemical heterogeneity. Infarct core volume and penumbra status are known to strongly influence both systemic inflammation and functional outcomes. Due to differences in imaging protocols and analysis methods between centers, standardized data for these variables were not available. We acknowledge this as a key limitation and suggest that future multicenter prospective studies with uniform imaging assessments are needed to validate the independent prognostic value of SIRI. Finally, the generalizability of our findings to other ethnic populations requires further validation.

## Conclusion

In conclusion, higher baseline Systemic Inflammation Response Index (SIRI) levels may be independently associated with an increased risk of poor 90-day functional outcomes after successful endovascular therapy with successful recanalization for large vessel occlusion stroke. SIRI may serve as a readily available biomarker associated with early functional outcomes and could have potential value in prognostic assessment, as its prognostic value may be more specific to functional recovery rather than hemorrhagic complications. Future prospective studies are needed to validate its utility and explore the value of dynamic monitoring.

## Data Availability

The original contributions presented in the study are included in the article/Supplementary material, further inquiries can be directed to the corresponding author.
