# Supplementary figures and images for: Prognostic value of systemic inflammation response index in successfully recanalized acute large vessel occlusion stroke patients: a retrospective study
Source: Front Neurol. 2026 May 28;17:1749452. doi: 10.3389/fneur.2026.1749452 (PMC13253426; doi:10.3389/fneur.2026.1749452)

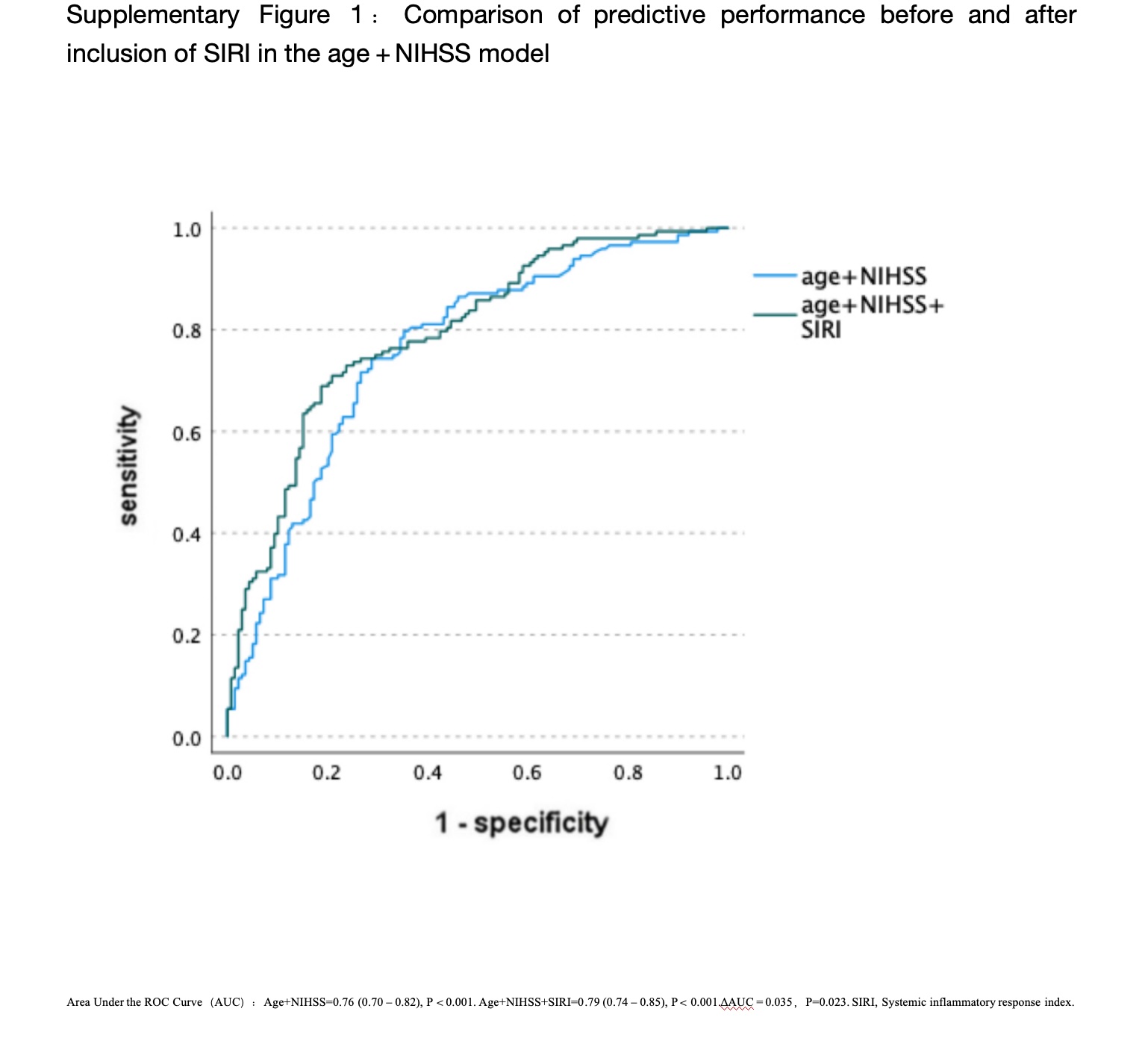

Supplement: Supplementary file 1 [file Image_1.jpeg]

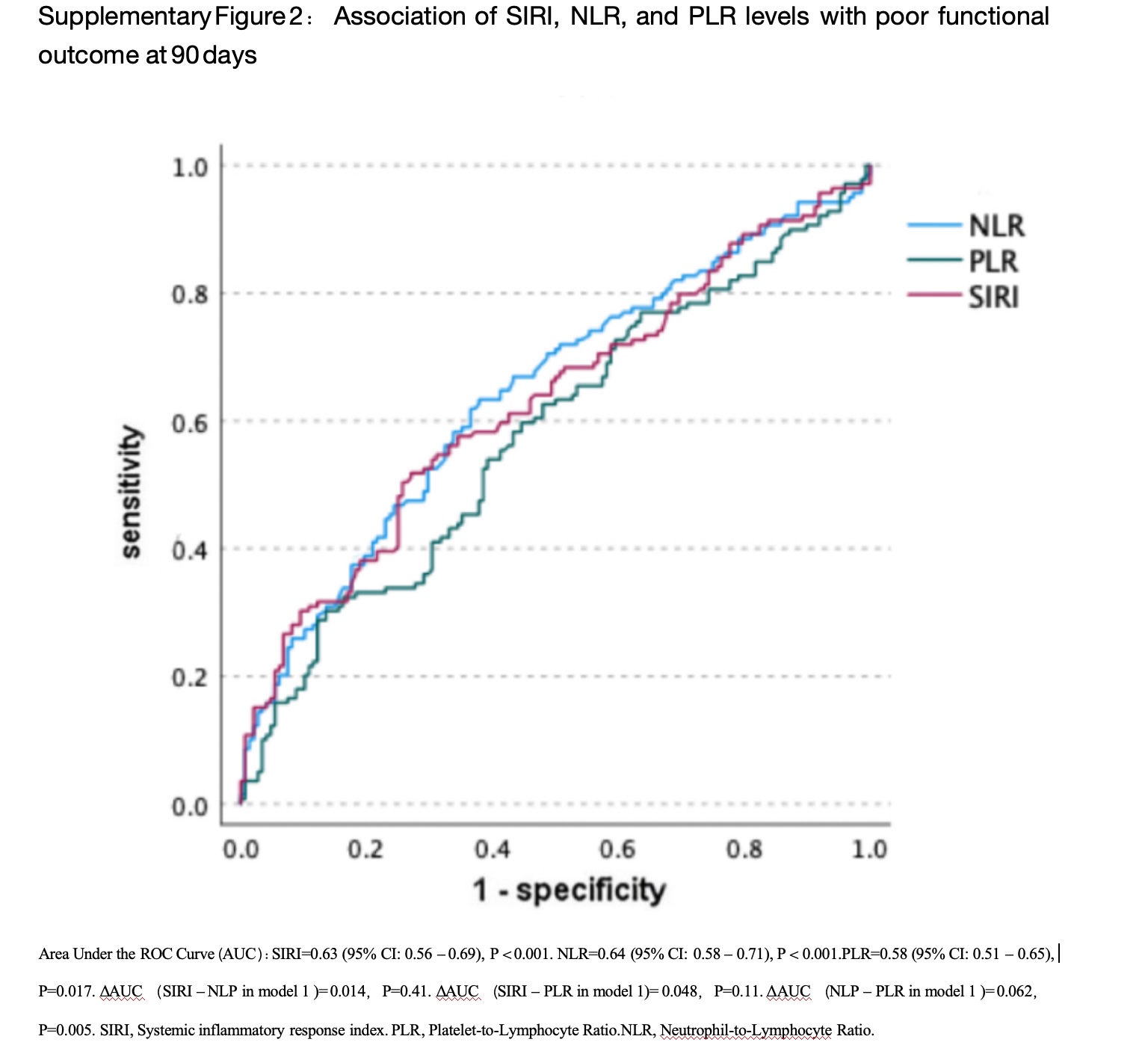

Supplement: Supplementary file 2 [file Image_2.jpeg]

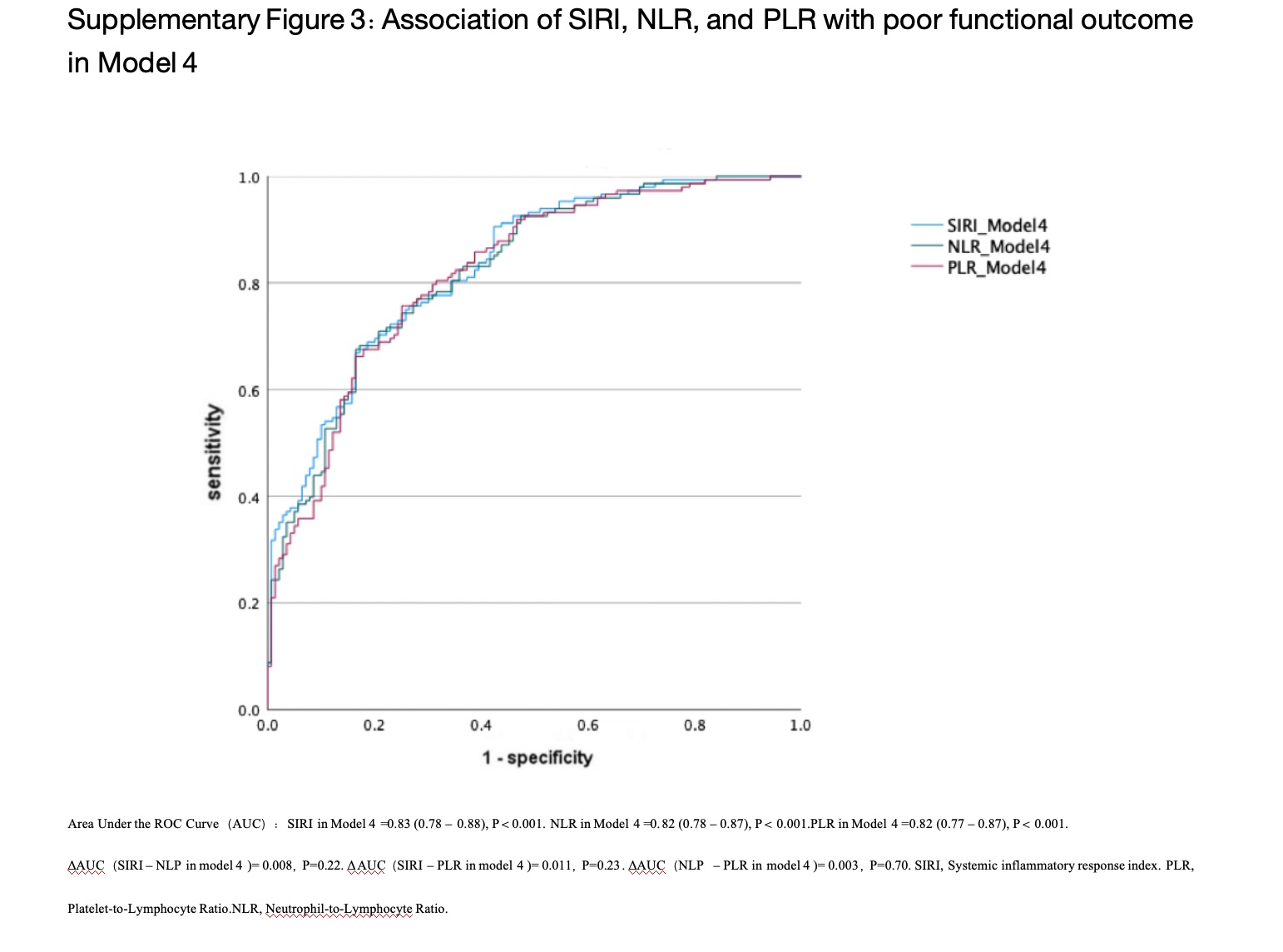

Supplement: Supplementary file 3 [file Image_3.jpeg]
