# Supplementary material for: Prognostic value of systemic inflammation response index in successfully recanalized acute large vessel occlusion stroke patients: a retrospective study
Source: Front Neurol. 2026 May 28;17:1749452. doi: 10.3389/fneur.2026.1749452 (PMC13253426; doi:10.3389/fneur.2026.1749452)
Supplement: Supplementary file 4 [file Table_1.docx]

Supplementary Table 1：Subgroup analysis of the association between baseline SIRI and 90‑day outcomes in patients with mild‑to‑moderate and severe stroke

|  |  | Model 1 | Model 2 | Model 3 | Model 4 |
| --- | --- | --- | --- | --- | --- |
|  | NIHSS | OR(95%CI)  P value | OR(95%CI)  P value | OR(95%CI)  P value | OR(95%CI)  P value |
| SIRI | NIHSS≤15 | 0.68(0.57－0.83)  <0.001^*^ | 0.65(0.50－0.85)  0.001^*^ | 0.63(0.48－0.83)  0.001^*^ | 0.63(0.47－0.85)  0.002^*^ |
|  | NIHSS＞15 | 0.56(0.35－0.90)  0.015^*^ | 0.52(0.30－0.90)  0.019^*^ | 0.66(0.17－1.16)  0.15 | 0.60(0.34－1.06)  0.08 |

Model 1, with no covariate adjustment. Model 2,adjusted for covariates with age, NIHSS score at admission. Model 3, adjusted for covariates with P < 0.1 (age, NIHSS score at admission, baseline blood glucose, smoking history, history of hypertension, diagnosis of atrial fibrillation after admission, receipt of bridging therapy, number of thrombectomy less than 4, OTP, OTR, anterior circulation, FIB). Model 4, adjusted for covariates with P < 0.05 (age, NIHSS score at admission, baseline blood glucose, history of smoking, history of hypertension, diagnosis of atrial fibrillation after admission, receipt of bridging therapy, number of thrombectomy less than 4, OTP, OTR). Multivariate Logistic regression analysis was used to calculate beta values and 95% confidence intervals. 95%CI, 95% confidence interval; OR, odds ratio; SIRI, Systemic inflammatory response index. * Marked represents P<0.05, indicating statistical significance.
