# Supplementary material for: Prognostic value of systemic inflammation response index in successfully recanalized acute large vessel occlusion stroke patients: a retrospective study
Source: Front Neurol. 2026 May 28;17:1749452. doi: 10.3389/fneur.2026.1749452 (PMC13253426; doi:10.3389/fneur.2026.1749452)
Supplement: Supplementary file 5 [file Table_2.docx]

|  |  | B | SD | β | t | P value | Multicollinearity Analysis | |
| --- | --- | --- | --- | --- | --- | --- | --- | --- |
|  |  |  |  |  |  |  | Tolerance | VIF |
| Model 5 | SIRI | -0.057 | 0.014 | -0.217 | -4.176 | ＜0.001^*^ | 0.993 | 1.007 |
|  | Age | -0.010 | 0.002 | -0.261 | -4.92 | ＜0.001^*^ | 0.958 | 1.004 |
|  | NIHSS | -0.024 | 0.004 | -0.284 | -5.368 | ＜0.001^*^ | 0.959 | 1.043 |
| Model 6 | SIRI | -0.035 | 0.026 | -0.134 | -1.322 | 0.187 | 0.321 | 3.116 |
|  | PLR | ＜0.001 | ＜0.001 | 0.032 | 0.385 | 0.701 | 0.484 | 2.065 |
|  | NLR | -0.022 | 0.017 | -0.163 | -1.323 | 0.187 | 0.216 | 4.622 |

Supplementary Table 2：Multicollinearity analysis of SIRI with age, NIHSS score at admission, NLR, and PLR

B,Unstandardized Regression Coefficient. SD, Standardized Regression Coefficient. β,Standardized Regression Coefficient.SIRI, Systemic inflammatory response index.PLR, Platelet-to-Lymphocyte Ratio.NLR, Neutrophil-to-Lymphocyte Ratio.* Marked represents P<0.05, indicating statistical significance.
